# Modelling Nonstationary Gene Regulatory Processes

**DOI:** 10.1155/2010/749848

**Published:** 2010-07-20

**Authors:** Marco Grzegorcyzk, Dirk Husmeier, Jörg Rahnenführer

**Affiliations:** ^1^Department of Statistics, TU Dortmund University, 44221 Dortmund, Germany; ^2^Biomathematics and Statistics Scotland, JCMB, The King's Buildings, Edinburgh EH93JZ, UK

## Abstract

An important objective in systems biology is to infer gene regulatory networks from postgenomic data, and dynamic Bayesian networks have been widely applied as a popular tool to this end. The standard approach for nondiscretised data is restricted to a linear model and a homogeneous Markov chain. Recently, various generalisations based on changepoint processes and free allocation mixture models have been proposed. The former aim to relax the homogeneity assumption, whereas the latter are more flexible and, in principle, more adequate for modelling nonlinear processes. In our paper, we compare both paradigms and discuss theoretical shortcomings of the latter approach. We show that a model based on the changepoint process yields systematically better results than the free allocation model when inferring nonstationary gene regulatory processes from simulated gene expression time series. We further cross-compare the performance of both models on three biological systems: macrophages challenged with viral infection, circadian regulation in *Arabidopsis thaliana*, and morphogenesis in *Drosophila melanogaster*.

## 1. Introduction

An important objective in systems biology is to infer regulatory networks from postgenomic data. Bayesian networks have been widely applied as a popular tool to this end—see, for example, [1]—and novel fast Markov chain Monte Carlo (MCMC) algorithms can be applied to systematically search the space of network structures for those that are most consistent with the data [2, 3]. One reason for the popularity of Bayesian networks has been the tractability of the marginal likelihood of the network structure. This term describes how well the model structure explains the data. Its computation is usually challenging, as it requires the solution of an integral over the entire parameter space. To obtain a closed-form expression, two probabilistic models with their respective conjugate prior distributions have been employed: the multinomial distribution with the Dirichlet prior (BDe) and the Gaussian distribution with the normal-Wishart prior (BGe). These approaches are restricted in that they either require a data discretisation (BDe: Bayesian Dirichlet equivalence score) or can only capture linear relationships (BGe: Bayesian Gaussian equivalence score). Recently, a generalisation of the BGe model based on a combination of a mixture model and the allocation sampler has been proposed [4], the Bayesian Gaussian Mixture (BGM) model. In the BGM model, data points are assigned to different compartments (subsets of the data) with the allocation sampler [5]. Model parameters (and their distributions) are allowed to differ between compartments, while information is shared among the compartments via a common network structure. Given the network structure each compartment is modelled separately and independently with the Gaussian BGe model. 

The present work proposes a modification of the BGM model, which is more suitable for dynamic gene regulatory networks and gene expression time series. The proposed model, which we refer to as the BGM_*D*_ model, replaces the free allocation model by a multiple changepoint process, as, for example, employed in [6], to divide the time series into segments and thereby take the time structure into account. 

In a preliminary study [7], we focused on synthetic data from small network domains. We found that the new model avoids spurious self-feedback loops if feedback loops, such as *X*(*t*) → *X*(*t* + 1) (e.g., in molecular biology: transcription factors regulating their own transcription), are not excluded from the analysis. In this paper, we present the mathematical details of the new BGM_*D*_ model in greater depth, and we demonstrate that BGM_*D*_ yields better inference results for real biological systems. To this end, we analyse two gene expression time series from macrophages and *Arabidopsis thaliana* and cross-compare the inference results of the BGM model and the new changepoint process BGM_*D*_ model. 

Furthermore, we combine both models (the free allocation model and the changepoint process) with discrete Bayesian network methodology and we compare the performance of both models on inferring the morphogenetical stages of muscle development in *Drosophila melanogaster* from binary gene expression time series. 

We note that our modelling paradigm is complementary to other recently proposed approaches. A nonlinear nondiscretised model based on heteroscedastic regression has been proposed in [8]. However, this approach no longer allows the marginal likelihood to be obtained in closed-form and requires a restrictive approximation (the Laplace approximation, that is, an approximation based on a 2nd order Taylor series approximation of the log likelihood) to be adopted. Another nonlinear model based on node-specific Gaussian mixture models has been proposed in [9, 10]. Again, the marginal likelihood is intractable. The authors resort to the Bayesian information criterion (BIC) of [11] for model selection, which is only a good approximation to the marginal likelihood in the limit of very large data sets. A more flexible approach based on changing graphs between changepoints has been proposed in [12, 13]. Conceptually, the assumption of changing networks is reasonable for some biological scenarios, like morphogenesis, where gene-regulatory processes have been measured over a long period of time. However, for cellular processes on a shorter time scale, it is questionable whether it is the network structure rather than just the strength of the regulatory interactions that changes with time. For example, it is not the principle ability of a transcription factor to potentially bind to the promoter of a gene and thereby initiate transcription (i.e., not the network structure), but the extent to which this happens (i.e., the regulatory interaction strength). We therefore argue that, especially for short time series, it is more appropriate to leave the network structure invariant among segments and to allow the interaction parameters to vary with time by modelling the time segments (between changepoints) separately and independently with the Gaussian BGe model. The systematic sharing of information among segments via a common network structure also reduces potential problems with model overflexibility and overfitting, which a more flexible approach that allows for different network structures is susceptible to when the time series are short.

## 2. Methods

### 2.1. The Dynamical BGe Network

*Dynamical Bayesian networks* (DBNs) are flexible models for representing probabilistic relationships among interacting variables *X*_1_,…, *X*_*N*_. The graph *𝒢* of a DBN describes the relationships between the variables (nodes), which have been measured at equidistant time points *t* = 1,…, *m*, in the form of conditional probability distributions. An edge pointing from *X*_*i*_ to *X*_*j*_ means that the realisation of *X*_*j*_ at time point *t*, symbolically: *X*_*j*_(*t*), is influenced by the realisation of *X*_*i*_ at time point *t* − 1, symbolically: *X*_*i*_(*t* − 1). Effectively there is a bipartite graph structure between two time steps *t* and *t* + 1 so that the acyclicity constraint—known from static Bayesian networks—is guaranteed to be satisfied. Therefore, in principle, each node can be its own parent node in DBNs. Such self-feedback loops *X*_*n*_(*t* − 1) → *X*_*n*_(*t*) model autocorrelations and it depends on the application whether such self-feedback loops should be valid edges or ruled out altogether to focus the inference on the interactions between genes. *π*_*n*_ = *π*_*n*_(*𝒢*) denotes the parent (node) set of node *X*_*n*_ in *𝒢*; that is, the set of all nodes from which an edge points to node *X*_*n*_ in *𝒢*. Given a data set **D**, where **D**_*n*,*t*_ and **D**_(*π*_*n*_,*t*)_ are the *t*th realisations *X*_*n*_(*t*) and *π*_*n*_(*t*) of *X*_*n*_ and *π*_*n*_, respectively, DBNs are based on the following homogeneous Markov chain expansion:

(1) $$\begin{array}{cc} & P\left(D\;|\;𝒢,\theta \right)\\ & \;\;=\overset{N}{\underset{n=1}{\prod }}\overset{m}{\underset{t=2}{\prod }}P\left(X_{n}\left(t\right)=D_{n,t}\;|\;\pi_{n}\left(t-1\right)=D_{\left(\pi_{n},t-1\right)},\theta_{n}\right), \end{array}$$

where ***θ*** is the total parameter vector, composed of subvectors ***θ***_*n*_. ***θ***_*n*_ specifies the *n*th local conditional distributions *P*(*X*_*n*_(*t*) | *π*_*n*_(*t* − 1), ***θ***_*n*_) in the factorisation. The BGe model specifies the distributional form *P*(**D** | *𝒢*, ***θ***) as multivariate Gaussian distribution, and it assumes a normal-Wishart distribution as prior distribution *P*(***θ*** | *𝒢*) [14]. The local probability distributions *P*(*X*_*n*_(*t*) | *π*_*n*_(*t* − 1), ***θ***_*n*_) are then given by conditional linear Gaussian distributions. For discrete data **D** the BDe model specifies the distributional form of the likelihood *P*(*D* | *𝒢*, ***θ***) as a set of multinomial distributions, and assumes a Dirichlet distribution as the prior *P*(***θ*** | *𝒢*) for the unknown parameters [15]. In principle, the BDe model yields a higher modelling flexibility than the BGe model, but BDe requires a data discretisation that usually incurs a substantial information loss. Under fairly weak conditions imposed on the parameter vector (prior independence and modularity) and the prior distribution *P*(***θ***) (conjugacy), so that *P*(***θ*** | *𝒢*) = ∏_*n*=1_^*N*^ *P*(***θ***_*n*_ | *π*_*n*_), where *π*_*n*_ = *π*_*n*_(*𝒢*), the parameters can be integrated out analytically. For both scoring metrics BGe and BDe [16], the marginal likelihood then satisfies the same expansion rule as the Bayesian network with fixed parameters:

(2) $$\begin{array}{c} P\left(D\;|\;𝒢\right)=\int P\left(D\;|\;𝒢,\theta \right)P\left(\theta \;|\;𝒢\right)d\theta =\overset{N}{\underset{n=1}{\prod }}\Psi \left(D_{n}^{\pi_{n}}\right), \end{array}$$

where

(3) $$\begin{array}{cc} \Psi \left(D_{n}^{\pi_{n}}\right) & =\int \overset{m}{\underset{t=2}{\prod }}P\left(X_{n}\left(t\right)=D_{n,t}\;|\;\pi_{n}\left(t-1\right)=D_{\left(\pi_{n},t-1\right)},\theta_{n}\right)\\ & \;\;\times P\left(\theta_{n}\;|\;\pi_{n}\right)d\theta_{n}, \end{array}$$

and **D**_*n*_^*π*_*n*_^ : = {(**D**_*n*,*t*_, **D**_*π*_*n*_,*t*−1_) : 2 ≤ *t* ≤ *m*} denotes the subset of the data pertaining to node *X*_*n*_ and its parent set *π*_*n*_. For the Gaussian BGe model the (local) factors Ψ(**D**_*n*_^*π*_*n*_^) in (3) can be computed in closed-form, according to (15) and (24) in Geiger and Heckerman [14]. For the discrete multinomial BDe model, the (local) factors can be computed in closed form according to (19) in [16].

### 2.2. Gaussian Mixture Bayesian Network Model

In the Gaussian BGe model, the local distributions *P*(*X*_*n*_(*t*) | *π*_*n*_(*t* − 1), ***θ***_*n*_) are conditional linear Gaussian distributions. We generalise the BGe model by the introduction of a latent allocation vector **V**, which assigns the data points to *K* different mixture components, where *K* is inferred from the data by applying changepoint birth and death moves, along the lines of the changepoint model (e.g., see [6, 17]). As in the BGM model [4], conditional on the latent vector **V**, a separate BGe score can be computed for each of the *K* mixture components. 

The allocation vector **V** of size *m* − 1 describes the allocation of the time points *t* = 2,…, *m* to the *K* components. **D** ^(*V*,*k*)^ denotes all time points that are allocated to component *k*. The posterior probability of *𝒢*, **V**, and *K* is proportional to the joint distribution

(4) $$\begin{array}{c} P\left(𝒢,V,K\;|\;D\right)=\frac{P\left(𝒢,V,K,D\right)}{P\left(D\right)}\propto P\left(𝒢,V,K,D\right), \end{array}$$

and the joint distribution can be factorised as follows:

(5) $$\begin{array}{c} P\left(𝒢,V,K,D\right)=P\left(K\right)P\left(V\;|\;K\right)P\left(𝒢\right)P\left(D\;|\;𝒢,V,K\right), \end{array}$$

where

(6) $$\begin{array}{c} P\left(D\;|\;𝒢,V,K\right)=\overset{K}{\underset{k=1}{\prod }}P\left(D^{\left(V,k\right)}\;|\;𝒢\right)=\overset{K}{\underset{k=1}{\prod }}\overset{N}{\underset{n=1}{\prod }}\Psi \left(D_{n}^{(V,k),\pi_{n}}\right), \end{array}$$

and **D**_*n*_^(*V*,*k*),*π*_*n*_^ : = {(**D**_*n*,*t*_, **D**_*π*_*n*_,*t*−1_) | *t* ∈ {2,…, *m*} : **V**(*t*) = *k*} denotes the realisations of node *X*_*n*_ and its parent set *π*_*n*_ for those time points that have been allocated to the *k*th component. It can be seen from these equations that **V** acts as a filter which divides the data **D** into *K* different compartments **D** ^(**V**,*k*)^ (*k* = 1,…, *K*), for which separate independent BGe scores can be computed in closed-form using (2) and (3). The BGM counterpart of (3) is given by:

(7) $$\begin{array}{cc} & \Psi \left(D_{n}^{(V,k),\pi_{n}}\right)\\ & \;\;=\int \underset{t:V\left(t\right)=k}{\prod }P\left(X_{n}\left(t\right)=D_{n,t}\;|\;\pi_{n}\left(t-1\right)=D_{\left(\pi_{n},t-1\right)},\theta_{n}\right)\\ & \;\;\;\;\times P\left(\theta_{n}\;|\;\pi_{n}\right)d\theta_{n}. \end{array}$$

For instance, if we have *m* = 11 time points and one changepoint between *t*_6_ and *t*_7_ so that **V** assigns the time points *t*_2_,…, *t*_6_ to the first and the remaining time points *t*_7_,…, *t*_11_ to the second segment, then separate local BGe scores are computed for the data subsets **D**_*n*_^(**V**,1),*π*_*n*_^ : = {(**D**_*n*,*t*_, **D**_*π*_*n*_,*t*−1_) | 2 ≤ *t* ≤ 6} and **D**_*n*_^(**V**,2),*π*_*n*_^ : = {(**D**_*n*,*t*_, **D**_*π*_*n*_,*t*−1_) | 7 ≤ *t* ≤ 11}, according to (15) and (24) in Geiger and Heckerman [14]. 

When a data compartment **D** ^(**V**,*k*)^ is empty, then we set the factors Ψ(**D**_*n*_^(**V**,*k*),*π*_*n*_^) equal to 1 (*n* = 1,…, *N*). For *P*(*𝒢*), we take a uniform distribution over all graph structures subject to a fan-in restriction of |*π*_*n*_| ≤ *ℱ*. For *P*(*K*), we take a truncated Poisson distribution with *λ* = 1 restricted to 1 ≤ *K* ≤ *K* ^⋆^ as prior. We note that the MCMC inference scheme, which we will discuss in Section 2.3, does not sample **V** directly, but is based on local modifications of **V** based on changepoint birth, death, and reallocation moves. That is, different from the free allocation in the BGM model [4], we change the assignment of data points to components via a changepoint process (e.g., see [6, 17]). This reduces the complexity of the allocation space substantially and incorporates our prior knowledge that adjacent time points are likely to be assigned to the same component. We will refer to the new changepoint BGM model as the BGM_*D*_ model. 

We identify *K* with *K* − 1 changepoints: *b*_1_,…, *b*_*K*−1_ on the continuous interval [2, *m*], and for notational convenience we introduce the pseudo-changepoints *b*_0_ = 2 and *b*_*K*_ = *m*. The observation at time point *t* is assigned to the *k*th component, symbolically **V**(*t*) = *k*, if *b*_*k*−1_ ≤ *t* < *b*_*k*_. Following Green [17], we assume that the changepoints are distributed as the even-numbered order statistics of *L* : = 2(*K* − 1) + 1 points *u*_1_,…, *u*_*L*_ uniformly and independently distributed on the interval [2, *m*]. The motivation for this prior, instead of taking *K* − 1 uniformly distributed points, is to encourage *a priori* an equal spacing between the changepoints, that is, to discourage mixture components that contain only a short compartment of the time series. We note that the even-numbered order statistics prior on the changepoint locations induces a prior distribution on the allocation vector **V**. Deriving a closed-form expression is involved but not required, as our MCMC inference scheme does not sample **V** directly. The inference is based on local modifications of **V**. In particular, we employ three different move types: (i) an additional component (*K* → *K* + 1) can be generated by setting a new changepoint on the interval [2, *m*] (changepoint birth move), (ii) the number of components can be decremented (*K* → *K* − 1) by removing one of the existing changepoints (changepoint death move), and finally, (iii) the allocation vector **V** can be changed without affecting the number of components *K* by changing the position of one of the existing changepoints (changepoint reallocation move). For the acceptance probabilities of these local moves, only *P*(**V** | *K*) ratios, which are straightforward to compute, are required.

### 2.3. Multinomial Mixture Bayesian Network Model

The discrete multinomial BDe model can be generalised in analogy to the continuous Gaussian Bge model. As before, the allocation vector **V** divides the data into *K* compartments, and each compartment of the data is modelled separately and independently with the multinomial BDe scoring metric. We note that closed-form solutions for the Ψ(·)-terms in (7) can be obtained with (19) in [16]. We will refer to the free individual allocation model BGM with the BGe score being replaced by the discrete multinomial BDe score as the Bayesian Discrete Mixture (BDM) model, and accordingly we will refer to the discrete BDe counterpart of BGM_*D*_ as the BDM_*D*_ model. We note that the BDM_*D*_ model is similar to the *n* *s* *B* *D* *e* model of Robinson and Hartemink [12], except that BDM_*D*_ leaves the inferred network structures invariant in time to allow for more information sharing among segments. We will employ the discrete counterparts BDM and BDM_*D*_ for an independent comparison of the suitability of the free allocation model and the changepoint process for inferring dynamic gene-regulatory processes (from discrete data).

### 2.4. MCMC Inference

We now describe an MCMC inference algorithm that can be used to obtain a sample {*𝒢* ^*i*^, **V** ^*i*^, *K* ^*i*^}_*i*=1,…,*I*_ from the posterior distribution *P*(*𝒢*, **V**, *K* | **D**). Our algorithm combines the structure MCMC algorithm for Bayesian networks [18] with the changepoint model (e.g., see [6, 17]), and draws on the fact that conditional on the allocation vector **V**, separate BGe scores *P*(**D** ^(**V**,*k*)^ | *𝒢*) can be computed for the *K* data compartments. Note that this approach is equivalent to the idea underlying the allocation sampler [5]. The resulting algorithm is effectively an RJMCMC sampling scheme in the discrete space of network structures and latent allocation vectors, where the Jacobian in the acceptance criterion is always 1 and can be omitted. With probability *P* = .5, we perform a traditional structure MCMC move on the current graph *𝒢* ^*i*^ and leave the latent vector **V** and the number of mixture components *K* unchanged, symbolically: **V** ^*i*+1^ = **V** ^*i*^ and *K* ^*i*+1^ = *K* ^*i*^. A new candidate graph *𝒢* ^*i*+1^ is randomly drawn out of the set of graphs *𝒩*(*𝒢* ^*i*^) that can be reached from the current graph *𝒢* ^*i*^ by deletion or addition of one single edge. The proposed graph *𝒢* ^*i*+1^ is accepted with probability

(8) $$\begin{array}{cc} & A\left({𝒢}^{i+1}\;|\;{𝒢}^{i}\right)\\ & \;=\min\;\left\{1,\frac{P\left(D\;|\;{𝒢}^{i+1},V^{i},K^{i}\right)}{P\left(D\;|\;{𝒢}^{i},V^{i},K^{i}\right)}\cdot \frac{P\left({𝒢}^{i+1}\right)}{P\left({𝒢}^{i}\right)}\cdot \frac{\left|𝒩\left({𝒢}^{i}\right)\right|}{\left|𝒩\left({𝒢}^{i+1}\right)\right|}\right\}, \end{array}$$

where |·| is the cardinality, and the marginal likelihood terms have been specified in (6). The graph is left unchanged, symbolically *𝒢* ^*i*+1^ : = *𝒢* ^*i*^, if the move is not accepted. We note that the network reconstruction will be based on the marginal posterior probabilities of the individual edges, which can be estimated for each edge from the MCMC sample *𝒢* ^1^,…, *𝒢* ^*I*^ by the fraction of graphs in the sample that contain the edge of interest

(9) $$\begin{array}{c} \hat{E_{i,j}}=\frac{1}{I}\overset{I}{\underset{k=1}{\sum }}I_{i,j}\left({𝒢}^{k}\right), \end{array}$$

where *I*_*i*,*j*_(·) is the indicator function with *I*_*i*,*j*_(*𝒢* ^*k*^) = 1 if there is an edge from *X*_*i*_ to *X*_*j*_ in *𝒢* ^*k*^. 

With the complementary probability 1 − *P* we leave the graph unchanged: *𝒢* ^*i*+1^ = *𝒢* ^*i*^, and perform a move on (**V** ^*i*^, *K* ^*i*^). We change the current number of components *K* ^*i*^ via a changepoint birth or death move, or the allocation vector **V** ^*i*^ by a changepoint reallocation move along the lines of the Reversible Jump Markov chain Monte Carlo algorithm (RJMCMC) algorithm [17]. 

The changepoint birth (death) move increases (decreases) *K* ^*i*^ by 1 and may also have an effect on **V** ^*i*^. The changepoint reallocation move leaves *K* ^*i*^ unchanged and may have an effect on **V** ^*i*^. If with probability (1 − *P*) a changepoint move on (*K* ^*i*^, **V** ^*i*^) is performed, we randomly draw the move type. Under fairly mild regularity conditions (ergodicity), the MCMC sampling scheme converges to the desired posterior distribution if the equation of detailed balance is fulfilled [17]. The condition of detailed balance implies that for each move a complementary move is defined, and that the acceptance probability depends on the proposal probability of the complementary move. The moves presented below are designed such that there is a unique complementary death move for each birth move and vice versa. Moreover, each reallocation move can be reversed by a single (complementary) reallocation move. The acceptance probabilities for these three changepoint moves (*K* ^*i*^, **V** ^*i*^)→(*K* ^*i*+1^, **V** ^*i*+1^) are of the following form [17]:

(10) $$\begin{array}{c} A=\min\;\left\{1,\frac{P\left(D\;|\;{𝒢}^{i},V^{i+1},K^{i+1}\right)}{P\left(D\;|\;{𝒢}^{i},V^{i},K^{i}\right)}\times R\times B\right\} \end{array},$$

where *R* = *P*(**V** ^*i*+1^ | K^*i*+1^)*P*(*K* ^*i*+1^)/*P*(**V** ^*i*^ | *K* ^*i*^)*P*(*K* ^*i*^) is the prior probability ratio, and *B* is the inverse proposal probability ratio. The exact form of these factors depends on the move type.

(i) For a changepoint reallocation (*r*), we randomly select one of the existing changepoints *b*_*j*_ ∈ {*b*_1_,…, *b*_*K*−1_}, and the replacement value *b*_*j*_^†^ is drawn from a uniform distribution on [*b*_*j*−1_, *b*_*j*+1_] where *b*_0_ = 2 and *b*_*K*_ = *m*. Hence, the proposal probability ratio is one, the prior probabilities *P*(*K* ^*i*+1^) = *P*(*K* ^*i*^) cancel out, and the remaining prior probability ratio *P*(**V** ^*i*+1^ | *K* ^*i*+1^)/*P*(**V** ^*i*^ | *K* ^*i*^) can be obtained from page 720 in Green's RJMCMC paper [17]

(11) $$\begin{array}{c} R_{r}=\frac{\left(b_{j+1}-b_{j}^{†}\right)\left(b_{j}^{†}-b_{j-1}\right)}{\left(b_{j+1}-b_{j}\right)\left(b_{j}-b_{j-1}\right)},\;\;B_{r}=1. \end{array}$$

If there is no changepoint (*K* ^*i*^ = 1) the move is rejected and the Markov chain is left unchanged.

(ii) If a changepoint birth move (*b*) on *K* ^*i*^ is proposed, the location of the new changepoint *b* ^†^ is randomly drawn from a uniform distribution on the interval [2, *m*]; the proposal probability for this move is *b*_*K*^*i*^_/(*m* − 2), where *b*_*K*^*i*^_ is the (*K* ^*i*^-dependent) probability of selecting a birth move. The reverse death move, which is selected with probability *d*_(*K*^*i*^+1)_, consists in discarding randomly one of the (*K* ^*i*^ − 1) + 1 = *K* ^*i*^ changepoints. The inverse proposal probability ratio is thus given by *B* = *d*_(*K*^*i*^+1)_(*m* − 2)/(*b*_*K*^*i*^_ *K* ^*i*^). The prior probability ratio is given by the expression at the bottom of page 720 in Green's RJMCMC paper [17] slightly modified to allow for the fact that *K* components correspond to *K* − 1 changepoints, and we obtain

(12) $$\begin{array}{c} R_{b}=\frac{P\left(K^{i}+1\right)}{P\left(K^{i}\right)}\frac{2K^{i}\left(2K^{i}+1\right)}{{\left(m-2\right)}^{2}}\frac{\left(b_{j+1}-b^{†}\right)\left(b^{†}-b_{j}\right)}{\left(b_{j+1}-b_{j}\right)},\\ B_{b}=\frac{d_{\left(K^{i}+1\right)}\left(m-2\right)}{b_{K^{i}}K^{i}}. \end{array}$$

For *K* ^*i*^ = *K* ^⋆^ the birth of a new changepoint is invalid and the Markov chain is left unchanged. 

We note that the proposal probabilities *b*_*K*_ and *d*_(*K*+1)_ for birth and death moves can be chosen as follows:

(13) $$\begin{array}{c} b_{K}=c\cdot \min\;\;\left\{1,\frac{P\left(K+1\right)}{P\left(K\right)}\right\},\\ d_{\left(K+1\right)}=c\cdot \min\;\;\left\{1,\frac{P\left(K\right)}{P\left(K+1\right)}\right\}, \end{array}$$

where *c* is a constant that can be chosen as large as possible subject to the constraint *b*_*K*_ + *d*_*K*_ ≤ 0.9 for *K* = 1,…, *K* ^⋆^. This choice yields both a certain acceptance rate of the MCMC sampling scheme [17] and a simple prior probability (Hastings) ratio. From *b*_*K*^*i*^_ · *P*(*K* ^*i*^) = *d*_(*K*^*i*^+1)_ · *P*(*K* ^*i*^ + 1) it follows that the ratio *d*_(*K*^*i*^+1)_/*b*_*K*^*i*^_ in the expression *R*_*b*_ cancels out against the prior ratio *P*(*K* ^*i*^ + 1)/*P*(*K* ^*i*^) in the expression *B*_*b*_, and the prior probability ratio simplifies to

(14) $$\begin{array}{c} R_{b}B_{b}=\frac{2\left(2K^{i}+1\right)}{\left(m-2\right)}\frac{\left(b_{j+1}-b^{†}\right)\left(b^{†}-b_{j}\right)}{\left(b_{j+1}-b_{j}\right)}. \end{array}$$

(iii) A changepoint death move (*d*) is the reverse of the birth move, and we obtain

(15) $$\begin{array}{c} R_{d}B_{d}=\frac{\left(m-2\right)}{2\left(2K^{i}-1\right)}\frac{\left(b_{j+1}-b_{j}\right)}{\left(b_{j+1}-b^{†}\right)\left(b^{†}-b_{j}\right)}. \end{array}$$

## 3. Data

We have evaluated the proposed BGM_*D*_ model on various synthetic data sets. For illustration purposes, we present results obtained for two studies with small networks where the nonlinearity was implemented by a sinusoidal transformation. In a second study, we focus on the two-gene expression time series from macrophages and *Arabidopsis thaliana*, which have been used for evaluating the BGM model. More details on the experimental settings can be found in the paper on BGM [4]. In a third study, we switch from the Gaussian BGe score to the discrete multinomial BDe score to infer discrete nonstationary time series from *Drosophila melanogaster*.

### 3.1. Small Synthetic Networks

The first synthetic network consists of two nodes *X* and *Y* and possesses two edges. Node *X* has a recurrent feedback loop, symbolically*X*(*t*) → *X*(*t* + 1), and *X* acts as a regulator of node *Y*, symbolically *X*(*t*) → *Y*(*t* + 1). We consider the scenario of a nonlinear regulatory influence that *X* exerts on *Y*, whereby we implement the nonlinearity by a sinusoid transformation of *X*(*t*)'s signal on *Y*(*t* + 1). The state-space equations are given by

(16) $$\begin{array}{cc} X\left(t\right) & =X\left(t-1\right)+c+c_{X}\cdot \varepsilon_{X}\left(t\right),\\ Y\left(t\right) & =\sin\;\left(X\left(t-1\right)\right)+c_{Y}\cdot \varepsilon_{Y}\left(t\right), \end{array}$$

where *c*, *c*_*X*_, and *c*_*Y*_ are constants, *ε*_*X*_(.), *ε*_*Y*_(.) are iid normally distributed random variables. 

The second synthetic network is a generalisation of the two node domain where three nodes *Y*_1_, *Y*_2_, and *Y*_3_ are regulated by *X*. The relationships are again realised by sinusoids, whereby we shift the periods: *Y*_*i*_(*t*) = sin (*X*(*t* − 1) + *τ*_*i*_ · *π*) + *c*_*Y*_ · *ε*_*Y*,*i*_(*t*) with *τ*_1_ = 0, *τ*_2_ = 2/3, and *τ*_3_ = 4/3. For both networks we set the drift term *c* = 2*π*/40 to ensure that (on average) the complete period [0,2*π*] of the sinusoid is involved, and we generate *m* = 41 observations for four different combinations of the coefficients *c*_*X*_ and *c*_*Y*_.

### 3.2. Bone Marrow Derived Macrophages

Interferons (IFNs) play a pivotal role in the innate and adaptive mammalian immune response against infection, and central research efforts therefore aim to elucidate their regulatory interactions [19]. For the present study, we have analysed gene expression time series from bone marrow-derived macrophages, which were sampled at *m* = 25 × 30 minute time intervals. The macrophages were subjected to three external conditions: (1) infection with Cytomegalovirus (CMV), (2) treatment with Interferon Gamma (IFN*γ*), and (3) infection with Cytomegalovirus after pretreatment with IFN*γ* (CMV+IFN*γ*). Samples derived from the macrophages were hybridised to Agilent mouse genome arrays. We focus on *N* = 3 Interferon-regulatory factors Irf1, Irf2, and Irf3. These factors are the key regulators in the response of the macrophage cell to pathogens. The macrophages data sets used in the study are publicly available from http://www.bioss.ac.uk/associates/marco/supplement/.

### 3.3. Circadian Regulation in *Arabidopsis thaliana*

We have also analysed two-gene expression time series from *Arabidopsis thaliana* cells, which were sampled at *m* = 13 × 2 hour time intervals with Affymetrix microarray chips. The expressions were measured twice independently under experimentally generated constant light condition, but differed with respect to the prehistories. In the first experimental scenario, *T*_20_, the plants were entrained in a 10 h : 10 h light/dark-cycle, while the plants in the second experimental setting, *T*_28_, were entrained in 14 h : 14 h light/dark-cycle. The analysis focuses on *N* = 9 genes, namely LHY, CCA1, TOC1, ELF4, ELF3, GI, PRR9, PRR5, and PRR3, which are known to be involved in circadian regulation [20, 21]. The Arabidopsis data sets used in the study are publicly available from http://www.bioss.ac.uk/associates/marco/supplement/.

### 3.4. Muscle Development in *Drosophila melanogaster*

The gene expressions in *Drosophila melanogaster* cells were sampled at *m* = 67 time-steps during four different morphological stages of life: embryonic, larval, pupal, and adult stages. Since these phases cover time periods of different lengths, gene expression profiles were collected at nonequidistant time-points. The true morphological transitions occur at time points *t*_32_ (embryonic to larval), *t*_42_ (larval to pupal), and *t*_60_ (pupal to adult) [22]. Like other researchers [12], we focus our analysis on *N* = 13 genes involved in muscle growth and muscle development: EVE, GFL, TWI, MLC1, SLS, MHC, PRM, ACTN, UP, MYO61F, and MSP300. The quantile-discretised binary data set of these 13 Drosophila genes is available from Robinson and Hartemink [12].

## 4. Simulations and Evaluation

We impose a fan-in restriction of size *ℱ* = 3 on the maximal number of parent nodes per node as done in related articles [2, 4]. For the Gaussian BGe model the hyperparameters of the normal-Wishart prior were chosen maximally uninformative subject to certain regularity constraints [14]. In the second study, the hyperparameters of the discrete multinomial BDe model were chosen as explained in Heckerman and Geiger [16], whereby the total prior precision parameter was set to 1. 

We set *K* ^⋆^ = 10, and the burn-in and the sampling-phase of MCMC runs were set to 500,000 iterations each, and we sampled every 1,000 iterations during the sampling-phase. For each data set, we started 5 independent MCMC simulations from different initialisations, and we computed the potential scale reduction factor PSRF [23] based on the marginal edge posterior probabilities. As we observed, a sufficient degree of convergence for all data sets (PSRF < 1.2), we report only the results of the empty-seeded (graphs without any edges) MCMC runs. We note that each single MCMC simulation (even for the bigger domains Arabidopsis with *N* = 9 genes and Drosophila with *N* = 13 genes) was accomplished within a few hours using Matlab^©^ code on a SunFire X4100M2 machine with MAD Opteron 2224 SE dual-core processor. 

More generally, we note that the computational complexity in network structure space is $N\sum_{k=0}^{ℱ}\left(\begin{array}{c} N\\ k \end{array}\right)\sim O(N^{ℱ+1})$, where *N* is the number of nodes and *ℱ* is the maximum number of parent nodes. The complexity in changepoint configuration space is of order *m* ^*K*^, where *K* is the number of changepoints, and *m* is the number of time points. Hence, the problem is of polynomial complexity in *N* and *m*, and *not* exponential complexity (i.e., it is not NP-hard). Polynomial complexity—as opposed to exponential complexity—does not impose any principled restrictions on the network size. However, the practical feasibility will depend on varying factors, like the efficiency of the software implementation and the capacity of parallel clusters. It also depends on the information content of the data, as increasing the number of nodes with limited number of experimental replications will increase the intrinsic uncertainty of inference. The practical decision on how many nodes and how large a network to consider will therefore usually be based on some preliminary data analysis. 

We note that we allowed for self-feedback loops for the synthetic data only. For the three real applications to biological systems, we ruled out self-feedback loops altogether to enable direct comparability with the results reported for the BGM model [4]. 

If two independent data sets **D** and **D**_‡_ are available for a network domain, predictive probabilities of the models BGM and BGM_*D*_ can be estimated straightforwardly [4]. For example, having sampled {*𝒢* ^*i*^, **V** ^*i*^, *K* ^*i*^}_*i*=1,…,*I*_ from the posterior distribution *P*(*𝒢*, **V**, *K* | **D**) via the MCMC inference scheme described above, the predictive probability of BGM_*D*_ can be estimated by

(17) $$\begin{array}{c} P\left(D_{‡}\;|\;D,{\text{BGM}}_{D}\right)=\frac{1}{I}\overset{I}{\underset{i=1}{\sum }}\overset{K^{i}}{\underset{k=1}{\prod }}\Psi \left({𝒢}^{i},D_{‡}^{\left(V^{i},k\right)}\;|\;D^{\left(V^{i},k\right)}\right), \end{array}$$

where

(18) $$\begin{array}{cc} & \Psi \left({𝒢}^{i},D_{‡}^{\left(V^{i},k\right)}\;|\;D^{\left(V^{i},k\right)}\right)\\ & =\overset{N}{\underset{n=1}{\prod }}\int \underset{t:V^{i}\left(t\right)=k}{\prod }P\left(X_{n}\left(t\right)=D_{‡n,t}\;|\;\pi_{n}\left(t-1\right)=D_{‡(\pi_{n},t-1)},\theta_{n}\right)\\ & \;\;\;\;\;\times P\left(\theta_{n}\;|\;\pi_{n},D^{\left(V^{i},k\right)}\right)d\theta_{n}. \end{array}$$

That is, when computing the BGe score for the compartment *D* ^(**V**^*i*^,*k*)^ with (6) and (7), the local prior distributions *P*(***θ***_*n*_ | *π*_*n*_) in (7) are replaced by posterior distributions *P*(***θ***_*n*_ | *π*_*n*_, **D** ^(**V**^*i*^,*k*)^). This results in a straightforward modification of the BGe score as follows. In (13) in Geiger and Heckerman [14], those training data that have been allocated to component *k*, symbolically **D** ^(**V**,*k*)^, are included in the conditioning part of the distribution, and the sufficient statistics are adjusted accordingly.

## 5. Results

### 5.1. Inference on Synthetic Data

**Figure 1:**
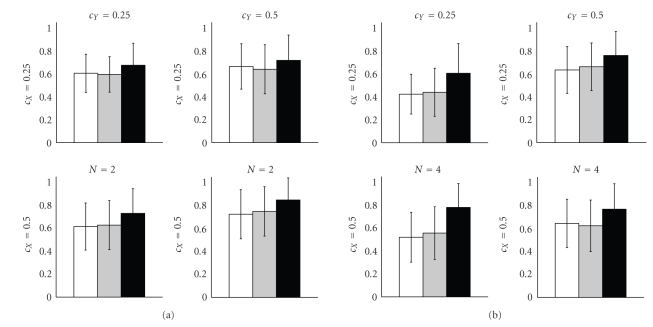
AUC histograms—Cross-method comparison on synthetic sine data. AUC scores for the synthetic sine network data with *N* = 2 nodes (a) and *N* = 4 nodes (b). The figure is laid out as a matrix, where rows and columns correspond to different noise levels *c*_*X*_ (rows) and *c*_*Y*_ (columns). In each histogram, the white bar shows the average AUC score for the BGe model. The grey bar shows the average AUC score of the BGM model, and the black bar shows the AUC score for the proposed BGM_*D*_ model. Each histogram shows averages and standard deviations obtained from 50 independent data instantiations.

For the synthetic sinusoid data, the true underlying network topologies are known so that the network reconstruction accuracy can be assessed via the area under the ROC (receiver operator characteristic) curve: AUC; this is a standard criterion that has been applied in numerous related articles (e.g., [24]). Figure 1 shows histograms of the average AUC scores obtained from the marginal edge posterior probabilities for the sinusoid networks with *N* = 2 and *N* = 4 nodes. Figure 1 is laid out as matrix, in which the rows and columns correspond to different *c*_*X*_ and *c*_*Y*_ coefficients (noise levels). We note that an increase of *c*_*X*_ reduces the autocorrelation of *X*, while increasing *c*_*Y*_ blurs the functional dependence of *Y*(*t* + 1) on *X*(*t*). The autocorrelation of *Y* is jointly influenced by both parameters. From the histograms it can be seen that the novel BGM_*D*_ model leads to a better network reconstruction accuracy in terms of average AUC values than the standard BGe model and the BGM model in the majority of scenarios. Further investigations showed that BGe and BGM yield lower AUC scores, since they tend to infer spurious self-feedback loops on node *Y* (*N* = 2) or on the nodes *Y*_*i*_ (*N* = 4), respectively.

**Figure 2:**
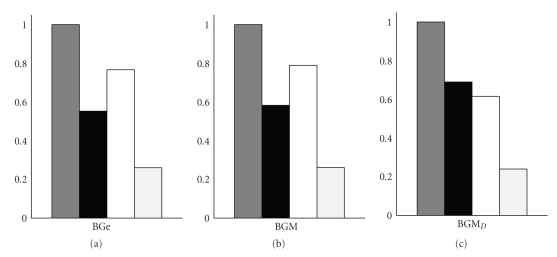
Edge Posterior Probabilities—Cross-method comparison on synthetic sine data. The figure shows three histograms of the inferred marginal edge posterior probabilities in the sinusoid network with *N* = 2 nodes and *c*_*X*_ = 0.5 and *c*_*Y*_ = 0.5 as obtained with BGe (a), BGM (b), and BGM_*D*_ (c). In each histogram, the four bars represent the four possible edges: Left: self-loop *X* → *X* (true); centre left: *X* → *Y* (true); centre right: self-loop *Y* → *Y* (false); right: *Y* → *X* (false). Each bar shows the average marginal posterior probability, averaged over 50 independent data instantiations. It is seen that BGe and BGM have a high propensity for learning the spurious feedback loop *Y* → *Y* (centre right white bars). BGM_*D*_ (right histogram) assigns a higher posterior probability to the correct edge *X* → *Y* (centre left black bar) and suppresses the spurious feedback loop *Y* → *Y* (centre right white bar)

This trend can be visualised by histograms of the average edge posterior probabilities. As an example, Figure 2 shows the average marginal edge posterior probabilities of the four possible edges for the *N* = 2 nodes sinusoid network with *c*_*X*_ = 0.5 and *c*_*Y*_ = 0.5. Consistently, all three models under comparison assign the highest posterior probability to the true self loop *X* → *X* and the lowest posterior probability to the false edge *Y* → *X*. But BGe and BGM favour the spurious feed-back loop *Y* → *Y* over the true edge *X* → *Y* while the proposed BGM_*D*_ suppresses the false self-feedback loop and assigns a higher edge posterior probability to the true edge *X* → *Y*. This shows that BGM_*D*_ yields a higher network reconstruction accuracy (see Figure 1), as it is less susceptible to inferring spurious self-feedback loops (see Figure 2).

### 5.2. Inference on Macrophages Data

**Figure 3:**
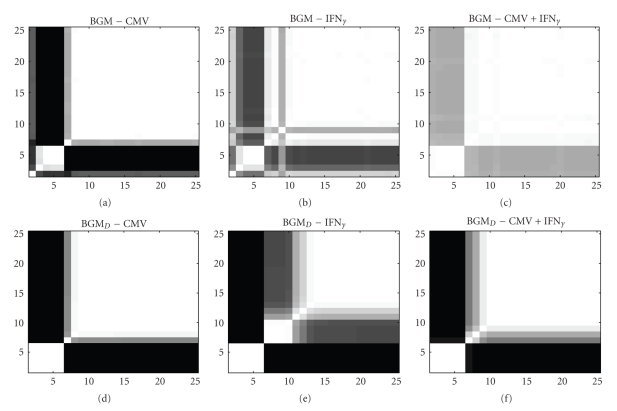
Heat maps—macrophages data. Graphical heat map presentation of the temporal connectivity structure for the macrophage gene expression time series. (a), (b), and (c): Heat matrices for experiments CMV (a), IFNG*γ* (b), and CMV+IFN*γ* (c) inferred with the BGM model. (d), (e), and (f): Heat matrices for experiments CMV (d), IFNG*γ* (e), and CMV+IFN*γ* (f) inferred with the novel BGM_*D*_ model. Each heat map indicates the estimated posterior probability of two time points being assigned to the same compartment (mixture component). The probabilities are represented by a grey shading, where white corresponds to a probability of 1, and black corresponds to a probability of 0. The numbers on the axes represent the time points of the time course experiment.

For the macrophages data the BGM model inferred a biologically plausible state change in the host macrophage brought about by infection (CMV) or immune activation (IFN*γ*), and a less pronounced state change in the combined condition CMV+IFN*γ* [4]. We compare these findings with results obtained with the novel BGM_*D*_ model. The fraction of sampled states for which two time points *t*_*i*_ and *t*_*j*_ are allocated to the same component *k* (1 ≤ *k* ≤ *K*) can be used as a connectivity measure *C*(*t*_*i*_, *t*_*j*_), and the resulting temporal connectivity structures are displayed graphically as heat maps in Figure 3. All six heatmaps in Figure 3 reflect the two-stage nature of the gene-regulatory processes in the host macrophages: the first part (time points (*t*_2_,…, *t*_6_)) and the last part of the three time series (time points (*t*_7_,…, *t*_25_)) are allocated to different components. For all three conditions, a stronger separation between the two regulatory states is inferred by the BGM_*D*_ model (see Figures 3(d), 3(e), and 3(f)). It appears that the BGM_*D*_ inference results are more consistent, as even for the combined condition (CMV+IFN*γ*) a clear trend towards a dichotomous regulatory process can be found (see Figure 3(d)). This finding (stronger separation) is consistent with our conjecture that the novel BGM_*D*_ assigns neighbouring time-points to the same compartment more likely a priori. Interestingly, the BGM inference outlier at time point *t*_9_ in Figure 3(b) yields a certain trend for a subdivision of the second compartment (*t*_7_,…, *t*_25_) by the BGM_*D*_ model. Instead of one outlying time point two substages (*t*_7_,…, *t*_10_) and (*t*_11_,…, *t*_25_) are inferred (see Figure 3(e)). To provide statistical evidence that the new BGM_*D*_ model does not overfit the data, we compute predictive probabilities for the BGM_*D*_ model and compare them with those reported for the BGM model [4].

To this end, we treat the three experiments as independent replications, and check via predictive probabilities whether the superiority of the proposed BGM_*D*_ model can be confirmed statistically. Table 1 gives the predictive probabilities reported in the BGM paper [4] and those obtained with the proposed BGM_*D*_ model. As the predictive probabilities for BGM_*D*_ are systematically better than those of BGM, we conclude that the BGM_*D*_ model yields more stable inference results, that is, a better generalisation performance.

### 5.3. Inference on *Arabidopsis thaliana* Data

**Figure 4:**
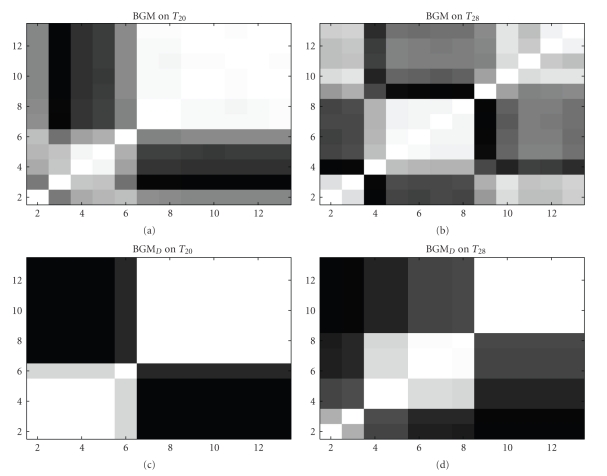
Heat maps. Arabidopsis data. Graphical heat map representations of the temporal connectivity structures for the *Arabidopsis thaliana* data. (a) and (b): heat matrices for experiments *T*_20_ (a) and *T*_28_ (b) inferred with the BGM model. (c) and (d): heat matrices for experiments *T*_20_ (c) and *T*_28_ (d) inferred with the novel BGM_*D*_ model. Each heat map indicates the posterior probability of two time points being assigned to the same compartment (mixture component). The probabilities are represented by a grey shading, where white corresponds to a probability of 1, and black corresponds to a probability of 0. The numbers on the axes represent the time points of the time course experiment.

For the *Arabidopsis thaliana* data, the BGM model also inferred a biologically plausible two-stage process [4]. In this application, the two stages are likely to be related to the diurnal nature of the dark-light cycle influencing the circadian genes. The plants were subjected to different prehistories, related to different lengths of the artificial, experimentally controlled light-dark cycle. The plants in experimental scenario *T*_28_ were entrained in an increased day length of 14 hours light followed by 14 hours darkness and in experiment *T*_20_ the plants were entrained in a decreased day length of 10 hours light followed by 10 hours darkness. As an effect of these two entrainments, a phase shift in the gene-regulatory processes between these two experiments was expected [4]. The BGM model inferred a certain trend for a phase shift of the changepoint (subjective day to subjective night) of about 4–6 hours as a consequence of the increased day length. The heat maps in Figures 4(a) and 4(b) show that the connected blocks (compartments) of the time series are shifted along the diagonal by 2-3 time-points (4–6 hours). The BGM_*D*_ model infers the same trend but with a stronger separation score between these compartments (see Figures 4(c) and 4(d)). We note that the BGM_*D*_ model is based on changepoints so that compartments once left cannot be revisited. That is, while the BGM model tends to allocate the first time points (*t*_2_, *t*_3_) and the last time points (*t*_9_,…, *t*_13_) in experiment T_28_ to one single component (light grey shading in the top right and bottom left area of the heat map in the top centre panel of Figure 4), the BGM_*D*_ model has to allocate the last time points (*t*_9_,…, *t*_13_) to an additional third component, as the first compartment (*t*_2_, *t*_3_) cannot be reused after the transition to the second compartment (*t*_4_,…, *t*_8_). 

**Figure 5:**
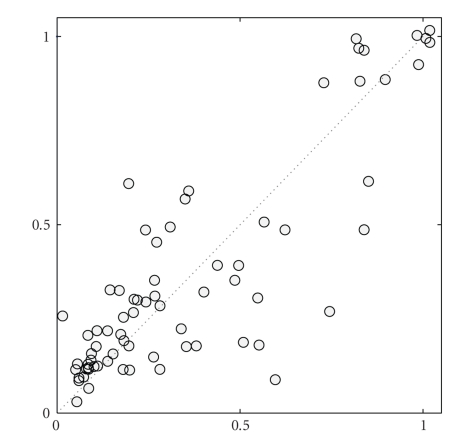
BGM_*D*_ scatter plot. Arabidopsis data. Scatter plot of the marginal edge posterior probabilities inferred with the proposed BGM_*D*_ model. In the plot the marginal edge posterior probabilities for time series *T*_20_: $\hat{E_{i,j}(T_{20})}$ (horizontal axis) are plotted versus the marginal edge posterior probabilities for time series *T*_28_: $\hat{E_{i,j}(T_{28})}$ (vertical axis).

As for the macrophages data, predictive probabilities can be computed by treating the two experiments *T*_20_ and *T*_28_ as independent replications. Table 2 gives the predictive probabilities reported for the BGM model [4] and those obtained with the new BGM_*D*_ model. In consistency with the results for the macrophages data, the resulting two predictive probabilities for BGM_*D*_ are better than those of the BGM model. A scatter plot $\hat{E_{i,j}(T_{20})}$ versus $\hat{E_{i,j}(T_{28})}$ of the inferred (marginal) posterior probabilities of the individual edges for the BGM_*D*_ model inference is given in Figure 5. As the individual edge posterior probabilities *E*_*i*,*j*_ do not differ drastically, we extract a network structure from the averaged probabilities: $\hat{E_{i,j}}=(\hat{E_{i,j}(T_{20})}+\hat{E_{i,j}(T_{28})})/2$. 

**Figure 6:**
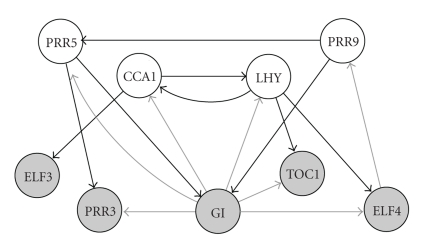
Predicted regulatory network of nine circadian genes in *Arabidopsis thaliana*. From the averaged marginal edge posterior probabilities (average of $\hat{E_{i,j}(T_{20})}$ and $\hat{E_{i,j}(T_{28})}$) of the proposed BGM_*D*_ model inference results a regulatory network can be extracted. Empty circles represent morning genes. Shaded circles represent evening genes. Edges indicate predicted interactions with an inferred marginal posterior probability greater than 0.5. Edges are black (grey) if they refer to a morning (evening) gene as regulator.

Figure 6 shows the gene interaction network that is predicted when keeping all edges with marginal posterior probability above 0.5. There are two groups of genes. Empty circles in the figure represent morning genes (i.e., genes whose expression peaks in the morning), shaded circles represent evening genes (i.e., genes whose expression peaks in the evening). There are several directed edges pointing from the group of morning genes to the evening genes such that each evening gene is regulated by at least one morning gene. Moreover, the two genes LHY and CCA1 seem to play a central role. This result is consistent with biological findings, where the morning genes were found to activate the evening genes, with LHY and CCA1 being central regulators [25]. Our reconstructed network also contains edges pointing into the opposite direction, from the evening genes back to the morning genes. This finding is consistent with biological observations [25], where the evening genes were found to inhibit the morning genes via negative feedback loops. In the reconstructed network, there are 9 edges (drawn in black) originating from the four morning genes while only 7 edges (drawn in grey) originate from the group of five evening genes. Biologically, this means that the activity of the morning genes is stronger than the activity of the group of evening genes and that the regulatory mechanisms are dominated by the morning genes in the network topology. This finding is consistent with the fact that following the light-dark cycle entrainment, the experiments were carried out in constant-light condition, resulting in a higher activity of the morning genes overall. Within the group of evening genes, the reconstructed network contains an edge between GI and TOC1. This interaction has been confirmed independently [26]. Hence, while a proper evaluation of the reconstruction accuracy is currently unfeasible—like many related studies, we lack a gold-standard owing to the unknown nature of the true interaction network—our study suggests that the essential features of the reconstructed network are biologically plausible and consistent with the literature.

### 5.4. Inference on *Drosophila melanogaster* Data

**Figure 7:**
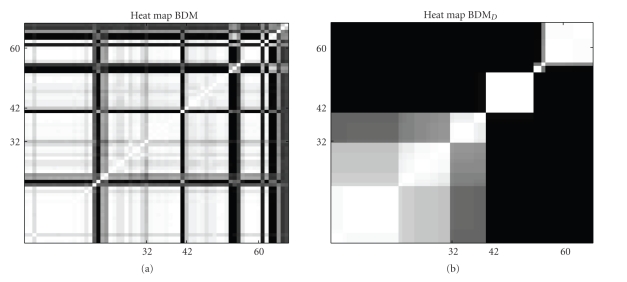
Heat maps for the discrete Drosophila data. Graphical heat map representations of the temporal connectivity structures for the *Drosophila melanogaster* data. (a) Heat matrix inferred with the BDM model. (b) Heat matrix inferred with the novel changepoint variant BDM_*D*_. Each heat map indicates the posterior probability of two time points being assigned to the same compartment (mixture component). The probabilities are represented by a grey shading, where white corresponds to a probability of 1, and black corresponds to a probability of 0. The numbers on the axes represent the time points of the time course experiment. Both axes have been ticked at the three real morphological stage transitions: embryonic to larval (*t*_31_ → *t*_32_), larval to pupal (*t*_41_ → *t*_42_), and pupal to adult (*t*_59_ → *t*_60_).

For an independent comparison of the free allocation model and the changepoint model, we carried out an analysis similar to Robinson and Hartemink [12] on the binary Drosophila muscle development gene expression time series. This time series can be analysed with the discrete counterparts BDM and BDM_*D*_ of our Gaussian Mixture models; see Section 2.3 for details. The graphical heat map representations in Figure 7 show that the BDM model does not infer the morphological stages of *Drosophila melanogaster*. Almost all time-points are strongly connected (white shading) and no separated blocks of connected time points have been inferred. Only a few time-points are allocated (as outliers) to other mixture components (black vertical and horizontal lines).

**Figure 8:**
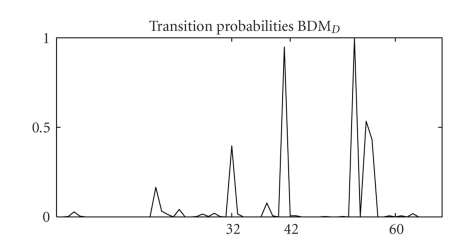
BDM_*D*_ transition posterior probabilities for Drosophila data. Graphical representation of the posterior probabilities of the changepoint locations inferred with the novel BDM_*D*_. The transition posterior probabilities (vertical axis) are plotted against the time axis (horizontal axis). The time axis has been ticked at the three real morphological stage transitions: embryonic to larval (*t*_31_ → *t*_32_), larval to pupal (*t*_41_ → *t*_42_), and pupal to adult (*t*_59_ → *t*_60_). We note that the BDM model is based on a free individual allocation of time points so that a transition posterior probability plot cannot be interpreted properly.

The novel BDM_*D*_ infers a clear block structure with different separated compartments. The connectivity structure corresponds well to the first two morphological transitions (i) embryonic to larval (*t*_31_ → *t*_32_) and (ii) larval to pupal (*t*_41_ → *t*_42_), whereby the separation between the embryonic and the larval stage is less pronounced (grey shading) than the separation between the larval and the pupal stage (black shading). The exact third morphological transition pupal to adult (*t*_59_ → *t*_60_) has not been inferred but it can be seen that two changepoints occur during the pupal stage before the third transition to the adult stage. Figure 8 shows a graphical presentation of the changepoint location posterior probabilities inferred with BDM_*D*_ and the same trends become obvious: the first two stage transitions have been inferred correctly while two further changepoints occur before the morphological stage transition from pupal to adult. We note that Robinson and Hartemink did find the same trends with their *n* *s* *B* *D* *e* model and they conclude that the third premature transition in the gene regulatory process can be explained biologically, since the gene expression program governing the transition from pupal to adult morphology should be active well before the time of the real morphological transition [12].

## 6. Discussion

Our empirical results have shown that the proposed BGM_*D*_ model performs consistently better than the BGM model. A possible explanation could be related to the latent space complexity of the models. For two components, we have *m* − 2 changepoint locations, but 2^*m*−1^ free allocations. This difference in latent space complexity gets aggravated for more components. The difference in performance between BGM_*D*_ and BGM could therefore be a consequence of the different degrees of convergence of the MCMC simulations. However, our convergence diagnostics based on potential scale reduction factors [23] did not indicate any significant difference in the convergence. It therefore appears that the higher latent space complexity of the BGM model does not adversely affect the MCMC convergence for gene expression time series of the length investigated in our study. This suggests that another explanation for the better performance of BGM_*D*_ over BGM has to be found. 

We will discuss that the performance difference is most likely a consequence of the different prior probabilities intrinsic to the models, which determine the factor *R* in (10). Since both models BGM and BGM_*D*_ employ the same Poisson distribution with parameter *λ* = 1 truncated to the interval [1, *K* ^⋆^] as prior for the number of mixture components, the difference between the two models BGM and BGM_*D*_ is imposed by the prior distribution of the allocation vector *P*(**V** | *K*). While the BGM model is based on a free allocation, the BGM_*D*_ model takes the time structure into account and employs a changepoint process. In this section we describe the differences between the two models in detail by three theoretical examinations, and these examinations reveal trends that appear to be immediately related to some of our empirical findings. 

In the first study, we compare the (temporal) connectivity structures that are introduced by the prior *P*(**V** | *K*). To this end, we infer the prior distribution *P*(*K*)*P*(**V** | *K*) of both models BGM and BGM_*D*_ via MCMC simulations that are purely prior-driven. That is, we employ an empty data set (without any data points) so that all acceptance probabilities depend on the prior probability ratios only, as *P*(**D** = *∅* | (*𝒢*, **V**, *K*)) = 1 for all combinations of *𝒢*, **V**, and *K*. Note that sampling network structures via edge operation moves on the graph *𝒢* becomes obsolete, because (i) the graphs do not have any effect on the likelihoods for an empty data set, and (ii) the graph priors cancel out in the prior probability ratio if a uniform graph prior *P*(*𝒢*) = const is used. We set *m* = 26; note that in this theoretical consideration based on empty data, *m* only determines the length of the allocation vector.

**Figure 9:**
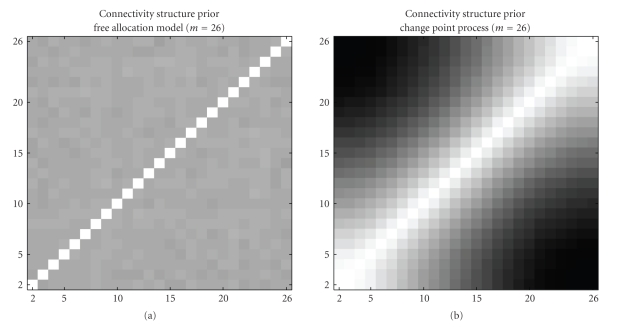
Prior connectivity structure for time series of length *m* = 26. Graphical heat map representations of the temporal connectivity structures imposed by the prior distribution *P*(*K*) · *P*(**V** | *K*) for *m* = 26 time points. (a) Free allocation model (BGM). (b) Changepoint process (BGM_*D*_). Each heat map indicates the prior probability of two time points being assigned to the same compartment. The probabilities are represented by a grey shading, where white corresponds to a probability of 1, and black corresponds to a probability of 0.5. The connectivity strengths were estimated from 10 independent MCMC simulations. In these simulations, an empty data set (without any data points) was used so that the inference was driven exclusively by the prior probability distribution *P*(*K*) · *P*(**V** | *K*).

After running 10 independent MCMC simulations with *m* = 26 to infer the prior distribution *P*(*K*) · *P*(**V** | *K*), we can compute the average prior connectivity strengths from the sampled allocation vectors. As before, the fraction of sampled allocation vectors for which two time points *t*_*i*_ and *t*_*j*_ are allocated to the same component*k* (1 ≤ *k* ≤ *K*) can be used as a connectivity measure *C*(*t*_*i*_, *t*_*j*_). Figure 9 shows heat maps of the inferred prior connectivity structure for the free allocation BGM model and the proposed changepoint process BGM_*D*_ model. The heat maps confirm our earlier conjecture that the proposed BGM_*D*_ model, which takes the time structure of the data into account, allocates neighbouring time points to the same compartment more likely a priori than the BGM model. More precisely, it can be seen from Figure 9(a) that the prior connectivity strength *C*(*t*_*i*_, *t*_*j*_) is the same for all *t*_*i*_ and *t*_*j*_ with *t*_*i*_ ≠ *t*_*j*_ in the BGM model. On the contrary, for the BGM_*D*_ model (Figure 9(b)), the connectivity strength *C*(*t*_*i*_, *t*_*j*_) decreases with the temporal distance between *t*_*i*_ and *t*_*j*_: for three time points *t*_*i*_, *t*_*j*_ and *t*_*k*_ with *t*_*i*_ < *t*_*j*_ < *t*_*k*_ we have *C*(*t*_*i*_, *t*_*j*_) < *C*(*t*_*i*_, *t*_*k*_). This finding explains why the proposed BGM_*D*_ model yields a stronger separation between the light : darkness induced stages in *Arabidopsis thaliana* (see Figure 4): the two-stage structure of gene-regulation in Arabidopsis is of a temporal form that is supported by the allocation vector prior *P*(**V** | *K*) of the BGM_*D*_ model. 

In the second and third theoretical study, we cross-compare the BGM model and the proposed BGM_*D*_ model in terms of prior probability ratios between the heterogeneous (nonstationary) and the homogeneous (stationary) state. That is, both models can be in the homogeneous state (i.e., the complete time series is modelled as one single compartment (*K* = 1)) or in a heterogeneous state (i.e., the time series is divided into *K* different compartments (*K* > 1)). For the BGM as well as the BGM_*D*_ model the prior probability ratio *R* between (i) the heterogeneous state consisting of two segments (*K* = 2) and (ii) the homogeneous state (*K* = 1) is given by

(19) $$\begin{array}{c} R=\frac{P\left(K=2\right)}{P\left(K=1\right)}\cdot \frac{P\left(V\;|\;K=2\right)}{1}, \end{array}$$

since *P*(**V** | *K* = 1) = 1 for both models. We consider the scenario where the time series is of length *m* and the allocation vector **V** divides the time series into two (non-empty) connected segments *t*_2_,…, *t*_*j*_ and *t*_*j*+1_,…, t_*m*_ (2 ≤ *j* ≤ *m*). The prior probability ratio of the BGM model is then given by

(20) $$\begin{array}{c} R_{\text{BGM}}=\frac{P\left(K=2\right)}{P\left(K=1\right)}\cdot \frac{\Gamma \left(\alpha_{0}\right)}{\Gamma \left(\alpha_{0}+\left(m-1\right)\right)}\cdot \overset{2}{\underset{k=1}{\prod }}\frac{\Gamma \left(\alpha_{k}+m_{k}\right)}{\Gamma \left(\alpha_{k}\right)}, \end{array}$$

where *α*_1_ = *α*_2_ = 1, *α*_0_ = *α*_1_ + *α*_2_ = 2, and *m*_*k*_ is the number of time points that have been allocated to the *k*th segment (*k* = 1,2); that is, *m*_1_ = *j* − 1 and *m*_2_ = *m* − *j* in our scenario. See [4] for details. The proposed BGM_*D*_ model requires a changepoint in the interval [*j*, *j* + 1] and it can be derived straightforwardly from (12):

(21) $$\begin{array}{c} R_{{\text{BGM}}_{D}}=\frac{P\left(K=2\right)}{P\left(K=1\right)}\cdot \overset{j+1}{\underset{j}{\int }}\frac{6\left(m-b_{1}\right)\left(b_{1}-2\right)}{{\left(m-2\right)}^{3}}db_{1}. \end{array}$$

**Figure 10:**
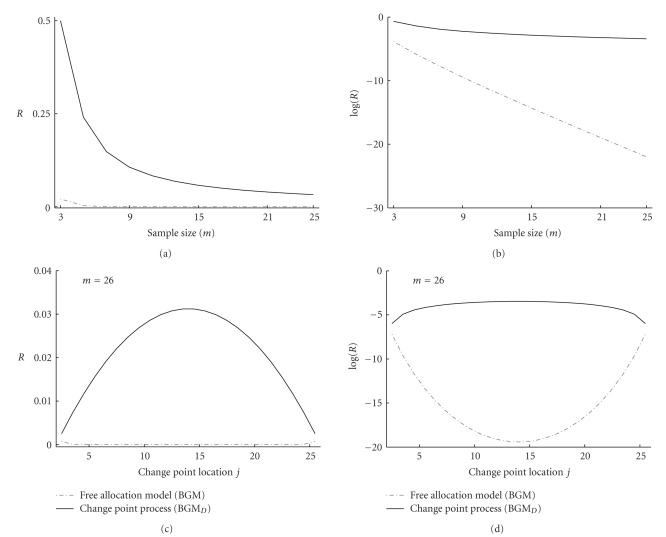
Prior probability ratios between the heterogeneous and the homogeneous state for (i) varying time series length *m* ((a) and (b)) and (ii) varying segment length proportions ((c) and (d)). (a) and (b): prior probability ratio *R* between (i) the heterogeneous state that consists of two equally-spaced segments *t*_2_,…, *t*_⌊*m*/2⌋+1_ and *t*_⌊*m*/2⌋+2_,…, *t*_*m*_ and (ii) the homogeneous state consisting of one single segment *t*_2_,…, *t*_*m*_. The prior probability ratios (vertical axis) are plotted in dependence on the time series length *m* = 3,5, 7,…, 25 (horizontal axis). The prior probability ratio was defined in (19). For clarity the logarithmic prior probability ratios are plotted in (b). (c) and (d): prior probability ratio *R* for a time series of length *m* = 26 between (i) the heterogeneous state with two segments *t*_2_,…, *t*_*j*_ and *t*_*j*+1_,…, *t*_*m*_ and (ii) the homogeneous state consisting of one single segment *t*_2_,…, *t*_*m*_ only. The prior probability ratios (vertical axis) are plotted in dependence on the changepoint location (horizontal axis). For the sake of clarity the logarithmic prior probability ratios are plotted in (b). See text for further details.

In the second theoretical study we vary the length of the time series *m* = 3,5, 7,…, 25, and we consider a heterogeneous time series consisting of two equally-spaced segments *t*_2_,…, *t*_⌊*m*/2⌋+1_ and *t*_⌊*m*/2⌋+2_,…, *t*_*m*_. This corresponds to *m*_1_ = *m*_2_ = 0.5 · (*m* − 1) in the BGM model. For the BGM_*D*_ model, we obtain with *j* = ⌊*m*/2⌋ + 1 that the changepoint has to be located in the interval *b*_1_ ∈ [*t*_⌊*m*/2⌋+1_, *t*_⌊*m*/2⌋+2_]. Figures 10(a) and 10(b) show the resulting (logarithmic) prior probability ratios in dependence on *m*. It can be seen that the prior ratio *R* for the BGM model is considerably lower than for the BGM_*D*_ model. Moreover, the logarithmic plot in Figure 10(b) shows that the prior ratio of the BGM model shows a much stronger decrease with the sample size *m* than the BGM_*D*_ model. This suggests that the BGM model imposes a more severe penalty for complexity (non-stationarity), which increases with increasing sample size *m*. This tendency may explain the finding in [4] for the macrophage gene expression time series, which we have reproduced in the present study (Figure 3(c)): the BGM model does not infer a clear two-phase nature of the time series under simultaneous immune activation (with IFN*γ*) and viral infection (with CMV). A possible biological explanation was offered in [4]. However, the novel BGM model does not support the hypothesis of a decreased probability for the two-phase nature (Figure 3(f)). Moreover, the previous analysis has revealed that a strong penalty against the two-phase process is inherent in the BGM model. This suggests that the results reported in [4], which we have reproduced in our study, might be an artefact of the BGM model rather than of genuine biological nature.

**Figure 11:**
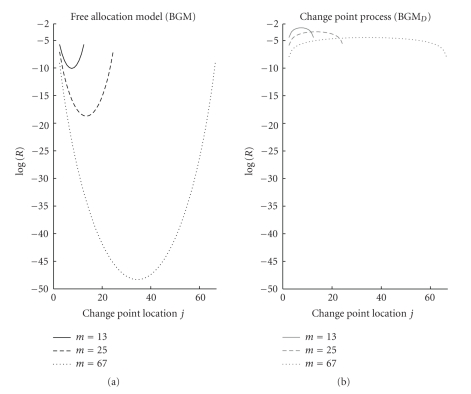
Prior probability ratios in dependence on both: the time series length m and the segment lengths proportions. Graphical representation of the prior probability ratio *R* between (i) the heterogeneous state consisting of two segments *t*_2_,…, *t*_*j*_ and *t*_*j*+1_,…, *t*_*m*_ and (ii) the homogeneous state consisting of one single segment *t*_2_,…, *t*_*m*_ only. The prior probability ratio was defined in (19). For three different lengths of the time series *m* = 13,25,67 the logarithmic prior probability ratios (vertical axis) between these two states are plotted in dependence on the changepoint location (horizontal axis). (a) BGM model. (b) BGM_*D*_ model.

In the third theoretical study, we fix the length of the time series *m* = 26 and we vary the last time point *j* = 2,3,…, 25 of the first segment in the heterogeneous state to illustrate the effect of unequal segment lengths *j* − 1 (*t*_2_,…, *t*_*j*_) and *m* − *j* (*t*_*j*+1_,…, *t*_*m*_). For the BGM model, this scenario yields *m*_1_ = *j* − 1 and *m*_2_ = *m* − *j*, and for the BGM_*D*_ model we have the changepoint location intervals *b*_1_ ∈ [*j*, *j* + 1], *j* = 2,3,…, 25. Figures 10(c) and 10(d) show the resulting prior probability ratios in dependence on the changepoint location *j*. The figure reveals contrary trends for the BGM and the BGM_*D*_ model. Figure 10(c) shows that the prior ratio of the BGM_*D*_ model peaks in the middle, that is for equally long time series segments, whereas it decreases monotonically with increasing asymmetry of the segment lengths. The BGM model (Figure 10(d)) exhibits the converse behaviour: the more disproportionate the segment lengths, the higher the prior ratio *R*. This behaviour becomes even more obvious when varying the time series length *m* and the breakpoint location *j* simultaneously. Figure 11 shows the logarithmic prior probabilities in dependence on both: the time series length *m* and the segment length proportions. For the BGM model (Figure 11(a)) the penalty term *P*(**V** | *K*) increases drastically with the sample size *m* if both segments are of a certain length. The higher the sample size the stronger the penalty for symmetric segment lengths. Only if *j* is either very low or very high, that is if the segment lengths are strongly disproportionate, does the size of the prior probability ratio *R* not change drastically with the sample size *m*. Figure 11(b) shows that the prior probability ratio of the proposed BGM_*D*_ model is less sensitive to both: the sample size *m* and the segment length proportions. We can conclude that it is the equability of the prior ratio of the proposed BGM_*D*_ that renders it superior when modelling nonstationary behaviour in long time series. That is, the penalty for dividing a time series into segments does not change drastically with the length of the time series *m*, and symmetric segment lengths are supported by the prior distribution *P*(**V** | *K*). On the contrary, the penalty term of the BGM model for dividing a time series into segments increases substantially with the length of the time series: the longer the time series, the lower the prior probability *P*(**V** | *K*). Only if the segment lengths are strongly asymmetric such that one segment is very long and the other very short, is the prior probability *P*(**V** | *K*) of a comparable size for different time series lengths *m*. This tendency provides a possible explanation for the failure of the BGM model on the *Drosophila melanogaster* gene expression time series. The heat map in Figure 7 shows that the proposed BGM_*D*_ model divides the time series into segments that are consistent with the morphogenesis of *Drosophila melanogaster*. The BGM model also tends to detect the correct segment boundaries. However, it then erroneously infers short segments consisting of a only few time points around these boundaries. This pattern is *not* consistent with the morphogenetical findings. The segmentation inferred with the BGM model thus suffers from artefacts that are an immediate consequence of what has been discussed above. Namely, that the BGM model penalises symmetric partitions much more strongly than asymmetric partitions and thereby encourages the formation of segments that consist of a single or only a few time points. Note that the BGM model becomes more susceptible to these artefacts as the length of the time series increases, as demonstrated in Figure 11(a). This explains why the difference between the BGM and the BGM_*D*_ model is less pronounced for the Arabidopsis and macrophage expression time series (Figures 3 and 4), which are considerably shorter (Arabidopsis: 13 time points, macrophages: 25 time points, Drosophila: 67 time points). The susceptibility of the BGM model to short-segment artefacts is not completely avoided here either, though, as can be seen from the heat map in Figure 3(b): While BGM_*D*_ infers three segments for the macrophages data under condition IFN_*γ*_ (see Figure 3(e)), BGM tends to allocate a single data point to a separate segment. 

On the synthetic sinusoid data, the proposed BGM_*D*_ model yields a higher network reconstruction accuracy than the BGM model, as the latter is more susceptible to inferring spurious self-feedback loops. This tendency can be explained from the previous mathematical analysis. Both the BGM and the BGM_*D*_ model effectively approximate the nonlinear function by a piecewise linear function. A good approximation of the sine wave requires three segments of approximately the same length, corresponding to the ascending, stationary and descending phase. As opposed to BGM_*D*_, the prior inherent to the BGM model heavily penalises against this equal-length segmentation (Figures 10 and 11); see the previous discussion. Now, from (16) it becomes clear that the data are strongly autocorrelated. More precisely, the *Y*(*t*)'s tend to exhibit a strong autocorrelation by virtue of the autocorrelation of the *X*(*t*)'s and the influence of *X*(*t*) on *Y*(*t* + 1). Given that the prior implicit in the BGM model impedes the proper piecewise linear model with equal-length segments, the BGM model tends to infer the second-best explanation of the data: explaining the realisation of the *Y*(*t*)'s via a direct modelling of the autocorrelation between the *Y*_*t*_'s themselves. This corresponds to (spurious) self-feedback loops. 

We note that the novel BGM_*D*_ model has been particularly designed for dynamic data with a temporal structure. The BGM model is not restricted to such data, and can equally be applied to both static (steady-state) and dynamic (time series) data. However, the greater flexibility of the BGM model and the intrinsic implications for the effective prior probability on segment lengths and numbers renders its application to time series data suboptimal. This suggests that the proposed BGM_*D*_ offers a useful new tool for the analysis of dynamic processes.

## 7. Conclusions

Two paradigms for relaxing the nonhomogeneity/nonlinearity restriction of dynamic Bayesian networks have been proposed in the literature: the changepoint process and the free allocation mixture model. The latter provides the proper approximation of a nonlinear regulation process by a piecewise linear process. The former provides a similar approximation, but under the assumption that the temporal processeses are sufficiently smooth, as the assignment of observations to mixture components of the model is done in the temporal domain rather than the domain of regulatory variables. It is obvious that inference in the free allocation model has a considerably higher computational complexity than the changepoint process. However, we have additionally discussed several principled shortcomings that are intrinsic to the methodology per se. We have proposed a new variation of the BGM model [4], which has turned out to be more suitable for the reconstruction of regulatory networks from nonstationary gene expression time series. Like the BGM model the new BGM_*D*_ model is based on a mixture model dividing the data points into compartments. The network structures are kept fixed among time series segments, and each segment is modelled separately and independently with the Gaussian BGe model for Bayesian networks. The methodological difference is that the BGM_*D*_ model employs a changepoint process to divide time series into segments instead of a free unrestricted allocation of data points. The practical inference follows the Bayesian paradigm and samples the network, the number of changepoints and the changepoint locations from the posterior distribution with Markov chain Monte Carlo (MCMC). 

In a first step, the inference problem was based on synthetic data from small network domains possessing self-feedback loops. Our empirical results show that the proposed BGM_*D*_ model suppresses spurious self-feedback loops and yields a higher network reconstruction accuracy than the standard BGe model or the BGM model. We also cross-compared the performance of the models on three real biological systems. On gene expression, time series related to (i) viral challenge of macrophages and (ii) circadian rhythms in Arabidopsis heat maps of the connectivity scores revealed that the new BGM_*D*_ model infers the biologically expected two-stage structures ((i) dichotomy between the healthy and diseased state of the cell and (ii) the diurnal contrast between light and darkness) more clearly than the BGM model. For assessing the statistical significance of the improvement we focused on predictive probabilities, and the proposed BGM_*D*_ model yields consistently higher scores than the BGM model. We extracted a gene regulatory network for the circadian clock-regulated genes in the *Arabidopsis thaliana* domain from the BGM_*D*_ inference results, and the reconstructed network shows features that are consistent with the biological literature.

Furthermore, for an independent comparison we combined the free allocation model and the changepoint process model with the discrete multinomial BDe scoring metric for Bayesian networks. Empirical results on binary gene expression time series related to muscle development in *Drosophila melanogaster* were consistent with the results from the first study on continuous data. The (discrete) changepoint process model (BDM_*D*_) infers a time series segmentation that is more consistent with the morphogenetical stages in *Drosophila melanogaster* than the free allocation model (BDM). 

We note that the ideal approach—from a biological point of view—for these three applications would be to use a supervised approach, for example, as described in Werhli and Husmeier [27], and to exploit the biological knowledge we have about the experimental conditions (e.g., the morphogenetical stages of Drosophila) for data segmentation before inference. However, we elected to use these data as a test case for evaluating the efficiency of unsupervised learning for the proposed changepoint-process DBN model. 

Finally, we cross-compared both models from a theoretical point of view. The theoretical study was based on a comparison of (i) the a priori imposed temporal connectivity structures and (ii) the prior probability ratios between the heterogeneous and the homogeneous states of the models. These results are consistent with our empirical findings, and lead to a deeper insight into the intrinsic difference between the two models.

## Figures and Tables

**Table 1:** Predictive Probabilities: Macrophages data. Logarithmic predictive probabilities for the macrophage data: log _*e*_ (*P*(**D**_‡_ | **D**)) for BGe, BGM (as reported earlier [4]) and the new BGM_*D*_ model. The standard deviations of the logarithmic probabilities are given in brackets. We note that the BGM values could be confirmed by our reanalysis of the data: the deviations were smaller than one standard deviation.

		**D**_‡_ = **D**_TEST_		
**D** = **D**_TRAIN_	*Model*	CMV	IFN*γ*	CMV and IFN*γ*
CMV	BGe	—	−76.01 (±0.07)	−45.26 (±0.03)
	BGM	—	−63.63 (±0.02)	−33.80 (±0.38)
	BGM_*D*_	—	−59.13 (±0.02)	−31.76 (±0.25)

IFN*γ*	BGe	−56.78 (± 0.05)	—	−57.30 (±0.05)
	BGM	−39.62 (± 0.02)	—	−42.69 (±0.11)
	BGM_*D*_	−34.08 (± 0.14)	—	−39.11 (±0.10)

CMV+IFN*γ*	BGe	−37.76 (±0.08)	−69.19 (± 0.06)	—
	BGM	−21.67 (±0.33)	−53.26 (± 0.51)	—
	BGM_*D*_	−18.58 (±0.09)	−51.11 (± 0.21)	—

**Table 2:** Predictive probabilities: *Arabidopsis thaliana* data. Logarithmic predictive probabilities for the *Arabidopsis thaliana* data: log _*e*_ (*P*(**D**_‡_ | **D**)) for BGe, BGM (as earlier reported [4]) and the new BGM_*D*_ model. The standard deviations of the logarithmic probabilities are given in brackets. We note that the BGM values could be confirmed by our reanalysis of the data: the deviations were smaller than one standard deviation.

**D**	*H*_*i*_	**D**_‡_ = *T*_20_	**D**_‡_ = *T*_28_
*T*_20_	BGe	—	−64.29 (±0.29)
	BGM	—	−53.69 (±0.42)
	BGM_*D*_	—	−52.39 (±0.78)

*T*_28_	BGe	−63.93 (±0.22)	—
	BGM	−54.78 (±0.63)	—
	BGM_*D*_	−48.69 (±0.32)	—
